# Supplementary material for: Gentianella lutescens subsp. carpatica J. Holub.: Shoot Propagation In Vitro and Effect of Sucrose and Elicitors on Xanthones Production
Source: Plants (Basel). 2021 Aug 11;10(8):1651. doi: 10.3390/plants10081651 (PMC8401808; doi:10.3390/plants10081651)
Supplement: Supplementary file 1 [file plants-10-01651-s001.zip › Figure S1.pdf]

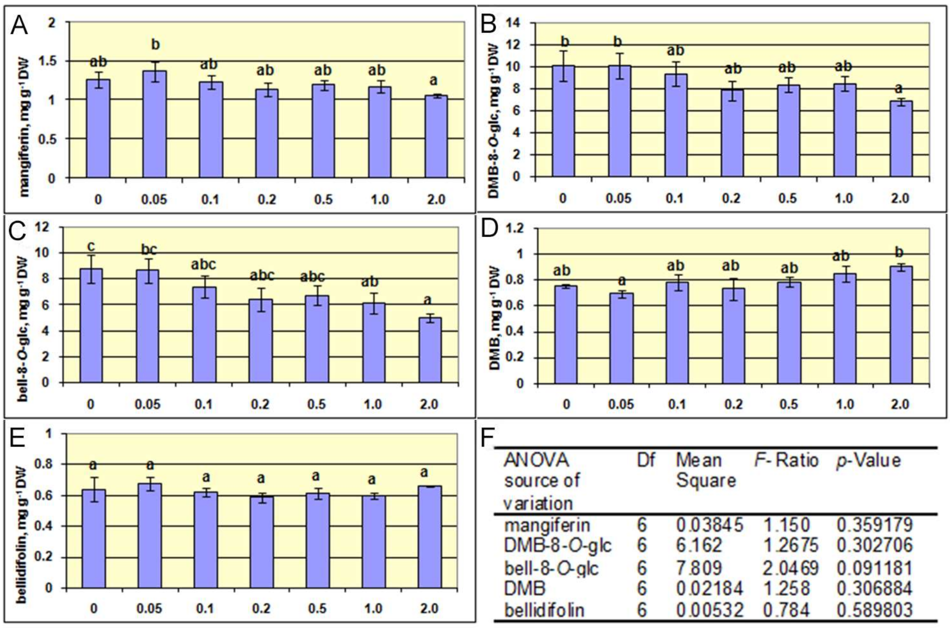

ANOVA source of variation	Df	Mean Square	*F*- Ratio	*p*-Value
mangiferin	6	0.03845	1.150	0.359179
DMB-8-*O*-glc	6	6.162	1.2675	0.302706
bell-8-*O*-glc	6	7.809	2.0469	0.091181
DMB	6	0.02184	1.258	0.306884
bellidifolin	6	0.00532	0.784	0.589803

Figure S1. The effect of increasing concentrations of BA (0–2 mg L^{-1}) on the content of xanthones mangiferin (A), demethylbellidifolin-8-*O*-glucoside (DMB-8-*O*-glc) (B), bellidifolin-8-*O*-glucoside (bell-8-*O*-glc) (C), demethylbellidifolin (DMB) (D), and bellidifolin (E) in shoots cultures of *G. lutescens* line 5 after 35 days of cultivation. Values are the means ± SE of six biological replicates (n = 6). Data were analysed by one-way ANOVA (F). Values followed by different letters are significantly different according to Fisher's LSD test at $p \leq 0.05$. .
