# Supplementary material for: Gentianella lutescens subsp. carpatica J. Holub.: Shoot Propagation In Vitro and Effect of Sucrose and Elicitors on Xanthones Production
Source: Plants (Basel). 2021 Aug 11;10(8):1651. doi: 10.3390/plants10081651 (PMC8401808; doi:10.3390/plants10081651)
Supplement: Supplementary file 1 [file plants-10-01651-s001.zip › Table S1.pdf]

Table S1. The results of nested ANOVA for the effects of increasing sucrose and sorbitol concentrations on the growth parameters (growth index, fresh and dry weight and % of dry matter) of shoot cultures of *G. lutescens* line 5. The bold values indicate statistically significant differences ($p \leq 0.05$).

ANOVA source of variation	Df	Mean Square	*F*- Ratio	*p*-Value
**Sucrose**				
Fresh weight	4	1285421	54.517	**0.000000**
Dry weight	4	2999	2.660	**0.044738**
Growth index	4	8.2287	54.831	**0.000000**
Dry matter %	4	0.05191	146.71	**0.000000**
Flowering	4	4.12000	4.54412	**0.003617**
**Sorbitol**				
Fresh weight	4	729024	18.993	**0.000000**
Dry weight	4	1723	1.030	0.405723
Growth index	4	4.8599	18.8620	**0.000000**
Dry matter %	4	0.027910	94.12	**0.000000**
