# Supplementary material for: Gentianella lutescens subsp. carpatica J. Holub.: Shoot Propagation In Vitro and Effect of Sucrose and Elicitors on Xanthones Production
Source: Plants (Basel). 2021 Aug 11;10(8):1651. doi: 10.3390/plants10081651 (PMC8401808; doi:10.3390/plants10081651)
Supplement: Supplementary file 1 [file plants-10-01651-s001.zip › Table S2.pdf]

Table S2. The results of two-way ANOVA for the influence of carbohydrate type (sucrose or sorbitol) and their concentrations on in vitro growth and flowering of shoots of *G. lutescens*. The bold values indicate statistically significant differences ($p \leq 0.05$).

Source	Sum of Squares	df	Mean Square	*F*-Ratio	*p*-Value
**Fresh weight, mg**					
(A) carbohydrate type	3074	1	3074	0.102	0.750739
(B) concentration	7471486	4	1867872	61.730	**0.000000**
A x B	359189	4	89797	2.968	**0.024209**
Error	2481220	82	30259		
**Dry weight, mg**					
(A) carbohydrate type	31	1	31	0.023	0.878964
(B) concentration	15695	4	3924	2.994	**0.023273**
A x B	5327	4	1332	1.016	0.403927
Error					
**Growth index**					
(A) carbohydrate type	0.1364	1	0.1364	0.687	0.409736
(B) concentration	49.3714	4	12.3429	62.145	**0.000000**
A x B	1.7203	4	0.4301	2.165	0.080115
Error	16.2864	82	0.1986		
**Dry matter, %**					
(A) carbohydrate type	16.19	1	16.19	7.10	**0.009299**
(B) concentration	1998.38	4	499.60	219.03	**0.000000**
A x B	64.24	4	16.06	7.04	**0.000063**
Error	187.04	82	2.28		
**Flowering**					
(A) carbohydrate type	17.60000	1	17.60000	35.37255	**0.000000**
(B) concentration	7.74417	4	1.93604	3.89107	**0.006065**
A x B	7.74417	4	1.93604	3.89107	**0.006065**
Error					
