# Supplementary material for: Gentianella lutescens subsp. carpatica J. Holub.: Shoot Propagation In Vitro and Effect of Sucrose and Elicitors on Xanthones Production
Source: Plants (Basel). 2021 Aug 11;10(8):1651. doi: 10.3390/plants10081651 (PMC8401808; doi:10.3390/plants10081651)
Supplement: Supplementary file 1 [file plants-10-01651-s001.zip › Table S3.pdf]

Table S3. The results of nested ANOVA for the effects of increasing sucrose and sorbitol concentrations on the xanthones production in shoot cultures of *G. lutescens* line 5. The bold values indicate statistically significant differences ($p \leq 0.05$).

ANOVA source of variation	Df	Mean Square	*F*- Ratio	*p*-Value
**Sucrose**				
mangiferin	4	10.5482	63.508	**0.000000**
DMB-8-*O*-glc	4	678.94	48.610	**0.000000**
bellidifolin-8-*O*-glc	4	102.753	28.460	**0.000000**
DMB	4	1.01244	9.2811	**0.000039**
bellidifolin	4	0.420090	5.5737	**0.001531**
**Sorbitol**				
mangiferin	4	0.14790	2.659	**0.049420**
DMB-8-*O*-glc	4	115.669	9.9284	**0.000020**
bellidifolin-8-*O*-glc	4	40.408	6.6653	**0.000450**
DMB	4	0.076329	7.5390	**0.000181**
bellidifolin	4	0.035898	2.5989	0.053411
