# Supplementary material for: Gentianella lutescens subsp. carpatica J. Holub.: Shoot Propagation In Vitro and Effect of Sucrose and Elicitors on Xanthones Production
Source: Plants (Basel). 2021 Aug 11;10(8):1651. doi: 10.3390/plants10081651 (PMC8401808; doi:10.3390/plants10081651)
Supplement: Supplementary file 1 [file plants-10-01651-s001.zip › Table S4.pdf]

**Table S4.** The results of nested ANOVA for the effects of elicitor type and concentrations on the shoot growth and xanthones production in shoot cultures of *G. lutescens* line 5. The bold values indicate statistically significant differences ($p \leq 0.05$).

| Source | Sum of Squares | df | Mean Square | *F*-Ratio | *p*-Value |
|---|---|---|---|---|---|
| **growth index** | | | | | |
| (A) elicitor type | 2.21915 | 2 | 1.10958 | 11.2014 | **0.000041** |
| (B) concentration | 1.84396 | 4 | 0.45541 | 4.5975 | **0.001893** |
| A x B | 2.94776 | 8 | 0.36826 | 3.7177 | **0.000767** |
| Error | 9.90566 | 100 | 0.09906 | | |
| **mangiferin** | | | | | |
| (A) elicitor type | 1.6776 | 2 | 0.8388 | 11.137 | **0.000085** |
| (B) concentration | 6.7056 | 4 | 1.6764 | 22.258 | **0.000000** |
| A x B | 1.8488 | 8 | 0.2311 | 3.068 | **0.006164** |
| Error | 4.2177 | 56 | 0.0753 | | |
| **DMB-8-*O*-glc** | | | | | |
| (A) elicitor type | 607.23 | 2 | 303.62 | 24.630 | **0.000000** |
| (B) concentration | 1508.37 | 4 | 377.09 | 30.591 | **0.000000** |
| A x B | 516.79 | 8 | 64.60 | 5.240 | **0.000065** |
| Error | 690.31 | 56 | 12.33 | | |
| **bell-8-*O*-glc** | | | | | |
| (A) elicitor type | 157.53 | 2 | 78.77 | 13.625 | **0.000015** |
| (B) concentration | 808.11 | 4 | 202.03 | 34.947 | **0.000000** |
| A x B | 149.61 | 8 | 18.70 | 3.235 | **0.004287** |
| Error | 323.73 | 56 | 5.78 | | |
| **DMB** | | | | | |
| (A) elicitor type | 1.911645 | 2 | 0.955822 | 52.5979 | **0.000000** |
| (B) concentration | 1.746994 | 4 | 0.436748 | 24.0338 | **0.000000** |
| A x B | 0.912565 | 8 | 0.114071 | 6.2772 | **0.000009** |
| Error | 0.999474 | 55 | 0.018172 | | |
| **bellidifolin** | | | | | |
| (A) elicitor type | 4.31979 | 2 | 2.15990 | 56.7827 | **0.000000** |
| (B) concentration | 3.09040 | 4 | 0.77260 | 20.3113 | **0.000000** |
| A x B | 2.42421 | 8 | 0.30303 | 7.9664 | **0.000001** |
| Error | 2.05405 | 54 | 0.03804 | | |
